# Influence of stress on physiological synchrony in a stressful versus non-stressful group setting

**DOI:** 10.1007/s00702-021-02384-2

**Published:** 2021-08-03

**Authors:** Bernadette Denk, Stephanie J. Dimitroff, Maria Meier, Annika B. E. Benz, Ulrike U. Bentele, Eva Unternaehrer, Nathalie F. Popovic, Wolfgang Gaissmaier, Jens C. Pruessner

**Affiliations:** 1 University of Konstanz, Konstanz, Germany; 2 Centre for the Advanced Study of Collective Behaviour, University of Konstanz, Konstanz, Germany; 3 Child- and Adolescent Research Department, Psychiatric University Hospitals Basel (UPK), University of Basel, Basel, Switzerland

**Keywords:** Physiological synchrony, Endocrine synchrony, Cortisol, Alpha-amylase, Stress contagion, Trier Social Stress Test for groups

## Abstract

Physiological synchrony (PS) is defined as the co-occurrence and interdependence of physiological activity between interaction partners. Previous research has uncovered numerous influences on the extent of PS, such as relationship type or individual characteristics. Here, we investigate the influence of acute stress on PS. We do so in a setting in which PS was not promoted, but contact between group members was explicitly minimized. We reanalyzed cortisol, alpha-amylase, and subjective stress data from 138 participants (mean age = $23.48 \pm 3.99$, 47.1% female) who previously underwent the Trier Social Stress Test for groups (TSST-G) or a non-stressful control task together, collected as part of a larger project by Popovic et al. (Sci Rep 10: 7845, 2020). Using a stability and influence model, an established method to test for synchrony, we tested whether individuals’ cortisol and alpha-amylase concentrations could be predicted by group members’ levels. We found cortisol PS in participants who were in the same group, the extent of which was stronger in the non-stressful control condition. For alpha-amylase, participants were synchronized as well; furthermore, there was an interaction between previous stress levels and PS. This suggests that while synchrony of both stress markers can occur in group settings even with spurious interaction, stressor exposure might attenuate its extent. We argue that if PS occurs in a sample where interaction was minimal, the phenomenon might be more widespread than previously thought. Furthermore, stressor exposure might influence whether a situation allows for PS. We conclude that PS should be investigated within group settings with various degrees of social interaction to further expose mechanisms of and influence on PS.

## Introduction

The co-occurrence and interdependence of changes in physiological reaction across interaction partners is called physiological synchronization [Ellamil et al. (2016); Palumbo et al. (2017), PS;], also referred to as empathic resonance or linkage. This cross-reactivity among group members or dyads has emerged as an important phenomenon to consider when investigating social processes. However, the mechanisms of PS have yet to be determined fully (Shamay-Tsoory et al. 2019; for an interesting theory, see Koban et al. 2019). An important question in this regard is under which circumstances PS happens (Gvirts and Perlmutter 2020). Here, type of relationship (Bizzego et al. 2020; Konvalinka et al. 2011), physical and social presence (Azhari et al. 2020; Järvelä et al. 2016), shared attitudes (Wróbel and Królewiak 2017), social context (Danyluck and Page-Gould 2019), shared movement (Gordon et al. 2020), and autism (McNaughton and Redcay 2020) have all been suggested to contribute to the phenomenon of synchronization across interaction partners.

In the current manuscript, we want to expand on this research and investigate the influence of acute stress on PS. While there have been some findings highlighting the importance of emotional state (Coutinho et al. 2019), e.g., whether acute stress actually increases or decreases PS remains unclear. To investigate this, here, we examine whether PS emerges in situations with strangers who have minimal interaction, and are even prevented from direct social contact with each other. For this purpose, we reanalyze a data set in which participants underwent a laboratory stressor [the Trier Social Stress Test for groups (TSST-G)], or a non-stressful control task together.

The TSST is a standardized paradigm to reliably elicit an endocrine stress response in a laboratory setting (Kirschbaum et al. 1993). The TSST-G (von Dawans et al. 2011) is similarly effective in stimulating cortisol release, but was designed to be more efficient by inducing stress in groups of up to six participants simultaneously. The TSST-G is by now widely employed as a successful laboratory stressor for groups (for a qualitative investigation, see Vors et al. 2018). During the TSST-G, participants are typically prohibited from visual contact. However, as part of the TSST-G participants complete two different oral presentation tasks, during which they listen to their fellow group members’ performances. Previous research has shown that tone of voice might indicate stress to others, and might be one pathway to PS [Dimitroff et al. (2017); Prochazkova and Kret (2017)].

If PS is present in our sample, and influenced by stressor exposure, this would indicate that PS emerges in situations which have previously not been regarded as promoting PS, the extent of which potentially varying based on psychological and physiological states. In addition to providing insights into possible mechanisms behind PS, our reanalysis can thus inform researchers employing the TSST-G on the possibility that PS is affecting individual participants’ stress responses.

In the original study, we exposed participants to the TSST-G or a non-stressful control task and measured cortisol and self-rated stress reactivity seven times to depict the change in physiological and subjective stress levels. For this reanalysis, we hypothesized that cortisol and alpha-amylase responses to the TSST-G within a group are not independent, i.e., that participants’ endocrine stress responses are at least partly explained by their group members’ cortisol trajectories. We also examine the influence of the stressor on this relationship, by comparing it to a non-stressful control condition. Finally, we explore group members’ influence on self-rated stress.

## Methods

To investigate cortisol and alpha-amylase synchronization during the TSST-G, we reanalyzed data from a larger project in which the TSST-G was employed and compared to a non-stressful control task. In our analysis, we made use of the information about which participants were tested together in one testing session (hereafter referred to as *group*). Additional details about the data collection as well as the aim of the larger project have been published previously (Popovic et al. 2020).

### Sample

*N* = 146 young healthy adults were recruited for the study. Of these, eight participants had to be excluded due to data loss or unclear group membership. Thus, the final sample in this analysis consisted of *N* = 138 participants (65 female, mean age = 23.48, and SD age = 3.99), with 75 participants (54.3%) in the TSST-G and 63 participants in the control condition. Participants were tested in 44 groups (mean group size = 3.14 participants, range 2–4; SD = 0.77). Testing took place between 9 am and 5 pm (median = 2 pm). Time of day was statistically controlled for to account for the circadian rhythm of the hypothalamic pituitary adrenal axis (Miller et al. 2016). Participants’ age, initial cortisol and alpha-amylase values, gender distribution, hormonal contraception intake, and group size did not differ between groups in the TSST-G condition compared to groups in the control condition (see Popovic et al. 2020, $p >.05$).

### Procedure

In the beginning of the experiment, participants gave written informed consent and subsequently filled out questionnaires. This anticipation phase took about 15 min. Afterward, participants were randomly assigned to a stress condition using the TSST-G or to a control condition. Following the experimental manipulation of stress levels, participants stayed together as a group, but worked independently on subsequent non-stressful tasks of risk perception which are not part of the current research question. The overall duration of the experiment was 85 min (for details, refer to Popovic et al. 2020).

### TSST-G and control task

Stress was induced using a slightly modified version of the TSST-G (see Popovic et al. 2020). The modifications made the protocol more feasible for testing groups of up to four participants. Participants were introduced to the task as a group. After a 5-min preparation phase, participants entered a room with two confederates acting in a non-emotional, non-supporting manner throughout the stress task, which included a public speaking task and a mental arithmetic task for a period of 4–6 min each, per participant. During both tasks, visual barriers prevented participants from having visual contact. In the control condition, groups performed similar tasks in writing and in absence of a committee (Popovic et al. 2020). The duration of the task was 27 min and the same in both experimental conditions, independent of the number of participants in the group.

### Endocrine measures

During the study, seven saliva samples were collected using Salivettes (Sarstedt AG & Co., Nümbrecht, Germany) in 12- to 15-min intervals, from 15 min before the onset of the TSST-G or control task to 45 min after. Cortisol levels (nmol/l) were determined using a time-resolved fluorescence immunoassay (Dressendörfer et al. 1992). Salivary alpha-amylase (U/ml) levels were determined using the enzyme kinetic method. The extraction of cortisol and alpha-amylase levels was performed by the biochemical laboratory of the University of Trier, Germany. For the analysis of cortisol, six additional participants had to be excluded from the study due to cortisol trajectories, suggesting that they were responding to a stressor happening prior to their arrival at the laboratory (high baseline with subsequently only declining levels) or because of very high cortisol levels throughout the entire experiment exceeding three standard deviations from the mean—see Popovic et al. (2020), for details. For the analysis of alpha-amylase, two participants had to be excluded due to all measures being zero.

### Self-rated stress

To assess psychological self-rated stress levels throughout the experiment, visual analogue scales ranging from 0 indicating no stress to 10 indicating maximal stress were used. Participants provided their assessments at the same time when saliva samples were taken. Self-rated stress measures will hereafter be referred to as *subjective stress*.

### Questionnaires

At the beginning of the experiment, participants filled out demographic questionnaires, as well as the Rosenberg Self-Esteem Scale (Rosenberg 1965), and the Perceived Stress Scale (Cohen et al. 1983). The Rosenberg Self-Esteem Scale is a measure for global self-esteem. The Perceived Stress Scale measures everyday stressful experiences. Both questionnaires were included in the design due to potentially confounding effects of physiological stress markers (see, Popovic et al. 2020). However, we found no such effects in our analysis.

### Statistical analysis

#### Cortisol synchrony

For the analysis of cortisol synchrony, we relied on a stability and influence model, a type of actor-partner interdependence model, following guidelines established by Thorson et al. (2018). In dyads, stability and influence models assess whether a partner’s (sender) previous measurement can explain variance in the other partner’s (receiver) current measurement, aka influence portion, beyond the receiver’s own previous measurement, aka stability portion (assessed through the autocorrelational structure). With each participant acting as sender as well as receiver, all possible combinations of dyads within a group were subsequently tested (for a group with participants A, B, and C, this would result in the combinations A–B, B–C, A–C, B–A, C–B, and C–A). We employed multi-level growth curve models to test for intraindividual stability and physiological synchrony. We included independent variables (fixed and random effects) stepwise into our model to predict cortisol values, using the following model equation (notation adapted from Finch and Bolin (2017)):

1 $$\begin{aligned} \begin{aligned}&y_{ti} ={} \beta _{00} + \beta _{10} * \mathrm {time}_{ti} + \beta _{20} * y_{(t-k)i} \\&\quad + \beta _{30} * \mathrm {condition}_i + \beta _{40} * S_{ti} \\&\quad +\beta _{50} * S_{(t-k)i} + U_{0i} + U_{1i} * \mathrm {time}_{ti} + e_{ti}, \end{aligned} \end{aligned}$$

where the dependent variable $y_{ti}$ represents the receiver’s cortisol level, for individual *i* at measurement point *t*, with $i = 1, ..., N$ and $t = 0, ..., T$. $\beta$s represent regression coefficients. Level-1 independent variables were linear, quadratic, and cubic effects of time (time$_t$ was the measurement point in minutes with index *t*), the preceding, or lagged, dependent variable $y_{(t-k)i}$ with lag size $k = 1\:(k = 1, 2, ..., M-1$), with *M* being the total number of measurements, and the sender’s concurrent and lagged cortisol values, $S_{ti}$ and $S_{(t-k)i}$. In contrast to the procedure described by Thorson et al. (2018), we also included the sender’s concurrent values as predictors as we expected to see simultaneous changes in sender and receiver. The level-2 independent variable was the group’s experimental condition (TSST-G versus control task). Random effects for each participant are represented by $U_{0i}$ (random intercept), and $U_{1i}$ (random slopes), where $U = {\mathcal {N}}(0, \sigma ^2_{U0})$. Random error is given by $e_{ti} = {\mathcal {N}}(0, \sigma ^2_e)$. For simplicity of presentation, polynomial time effects, covariates, as well as interaction effects were omitted in this notation, but were included in the analyses. Level-1 independent variables were centered, except for time effects (see Enders and Tofighi 2007). We did not use orthogonal coding of polynomial effects of the time variable based on suggestions by Biesanz et al. (2004).

In each step of our analysis, a more complex model was selected when its fit was significantly increased compared to the simpler model, as determined by analyses of variance and the Akaike Information Criterion (AIC). For the final model, regression coefficients were obtained (beta-coefficients and *F* values), and significance was determined. Furthermore, we calculated the coefficient of determination and tested model assumptions, including homogeneity of variance and normal distribution of residuals.

To test the influence of lag size on PS, as well as for illustrative purposes, we further calculated cross-correlation functions for all pairs of participants. Cross-correlation functions indicate the relationship between two time series dependent on lag size *k*. If $k \ne 0$, this means that values of one partner correlate with previous ($k < 0$) or future ($k > 0$) values of the other partner, i.e., during the interaction, one partner is lagging behind. While we only examined $k = 0$ and $k = 1$ in our model, significant relationships for all lag sizes $k = 0, 1, ..., M-1$ can be visualized.

Cross-correlation coefficients (CC coefficients) can be interpreted analogous to correlation coefficients. The significance of CC coefficients can be tested by comparing them to the conventional limit for significance (CCSL; see Dean and Dunsmuir 2016).

For illustrative purposes, we calculated the area under the curve with respect to the increase (AUC$_I$) for each participant’s cortisol time series. The AUC$_I$ indicates the overall increase and decrease of cortisol over time, resulting in one value per participant. Positive values indicate an overall increase from the starting point (i.e., the first measurement), while negative values indicate an overall decrease in cortisol. AUC$_I$ was calculated according to a formula provided by Pruessner et al. (2003).

#### Further analyses

While we mainly focused on cortisol synchrony, we also calculated PS of alpha-amylase, and synchrony of subjective stress. For the analysis of alpha-amylase and subjective stress, respectively, the same model as for cortisol was employed. In each model, covariates were included when they were significantly correlated with the respective dependent variable and, thus, differed for models of cortisol, alpha-amylase, and subjective stress.

#### Software

All statistical analyses were conducted in R version 3.6.2 (R Core Team 2019) with RStudio version 1.1.463 (RStudio Team 2020). Multi-level models were calculated using the *nlme* package (Pinheiro et al. 2019). The final model’ coefficient of determination was calculated using the *performance* package (Lüdecke et al. 2020).

## Results

**Fig. 1:**
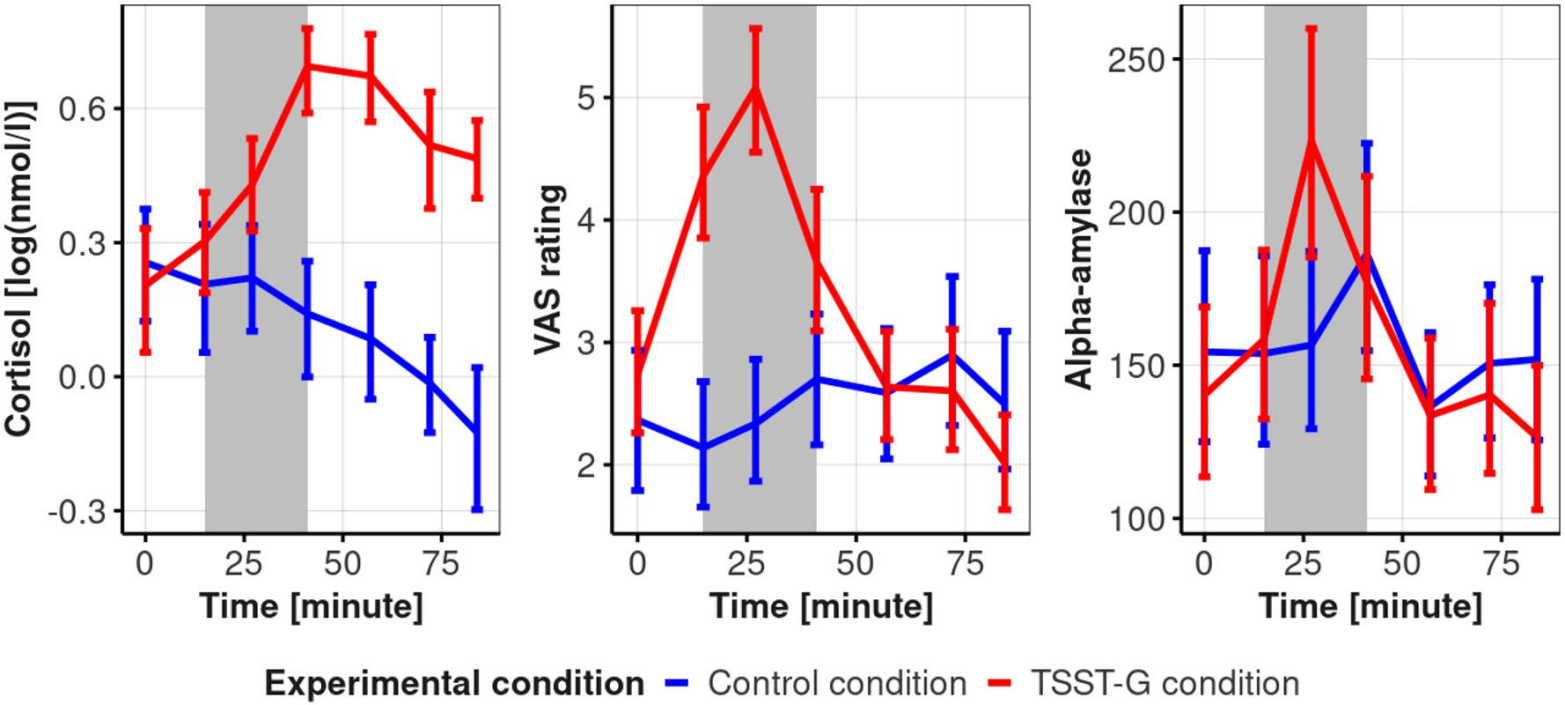
Cortisol (left), subjective stress (middle), and alpha-amylase (right) trajectories, mean, and standard errors for both experimental conditions. The gray rectangle indicates the time span of the intervention. Time = minutes since the first stress assessment.

**Fig. 2:**
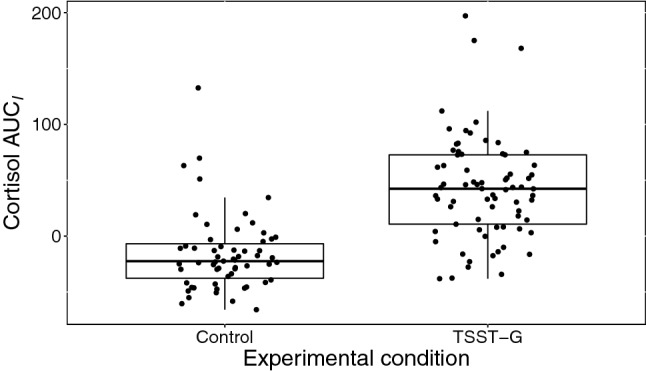
Boxplot of AUC$_I$ for cortisol in each experimental condition. While conditions differ significantly in overall cortisol responses, there is also pronounced variation within each condition

Figure 1 displays trajectories of cortisol concentration, alpha-amylase concentration, and subjective stress levels within each experimental condition. Despite the pronounced influence of the experimental condition, there was also considerable variance within each condition. For cortisol, the variation in AUC$_I$ values is shown in Fig. 2.

### Cortisol synchrony

Our hypothesis regarding PS during the TSST-G was tested using a multi-level stability and influence model. For cortisol, model fit was improved, compared to a basic model, when adding a random intercept for each participant, indicating inter-individual differences in baseline cortisol (intraclass coefficient ICC = 0.72). Model fit was further improved by adding a random linear, quadratic, and cubic time effect, indicating differences in individual cortisol trajectories. By adding all fixed effects that further improved overall model fit, we arrived at our final model, for which regression coefficients and their significance were evaluated.

Here, cortisol values changed significantly over time (linear effect: $F(1,1564) = 45.51, \:p < 0.001$; quadratic effect: $F(1,1564) = 111.81, \:p < 0.001$; and cubic effect: $F(1,1564) = 18.51, \:p < 0.001$). Overall, cortisol levels were higher in the TSST-G compared to the control condition ($F(1,129) = 8.89, \:p = 0.003$), with cortisol trajectories differing between experimental conditions (linear effect: $F(1,1564) = 129.84, \:p < 0.001$; quadratic effect: $F(1,1564) = 76.43, \:p < 0.001$; and cubic effect: $F(1,1564) = 9.55, \:p = 0.002$). Within-person stability explained further variance in cortisol levels ($F(1,1564) = 144.23, \:p < 0.001$), and changed over time (linear: $F(1,1564) = 508.87, \:p < 0.001$; quadratic: $F(1,1564) = 126.81, \:p < 0.001$; cubic: $F(1,1564) = 76.02, \:p < 0.001$). Within-person stability was slightly higher in the TSST-G condition overall ($F(1,1564) = 12.28, \:p < 0.001$); however, within-person stability decreased during the measurement after the experimental manipulation in the TSST-G condition (linear: $F(1,1564) = 0.68, \:p = 0.411$; quadratic: $F(1,1564) = 11.86, \:p < 0.001$; cubic: $F(1,1564) = 20.96, \:p < 0.001$). When examining PS, i.e., mutual influence between group members, we found a main effect of senders’ concurrent cortisol ($F(1,1564) = 8.30, \:p = 0.004$), but not lagged cortisol (i.e., influence of previous values; $F(1,1564) = 0.71, \:p = 0.400$). Senders’ concurrent influence did not change over time (all $p > 0.05$), but depended on experimental condition ($F(1,1564) = 7.28, \:p = 0.007$), with stronger PS in the control condition. Senders’ lagged influence was also stronger in the control condition ($F(1,1564) = 16.87, \:p < 0.001$), and changed over time (linear effects: $F(1,1564) = 0.55, \:p = 0.49$; quadratic effects: $F(1,1564) = 0, \:p = 0.99$; cubic effects: $F(1,1564) = 15.74, \:p < 0.001$), especially in the TSST-G condition (linear effects: $F(1,1564) = 1.4, \:p = 0.237$; quadratic effects:$F(1,1564) = 14.94, \:p < 0.001$; cubic effects: $F(1,1564) = 2.39, \:p = 0.122$).

**Fig. 3:**
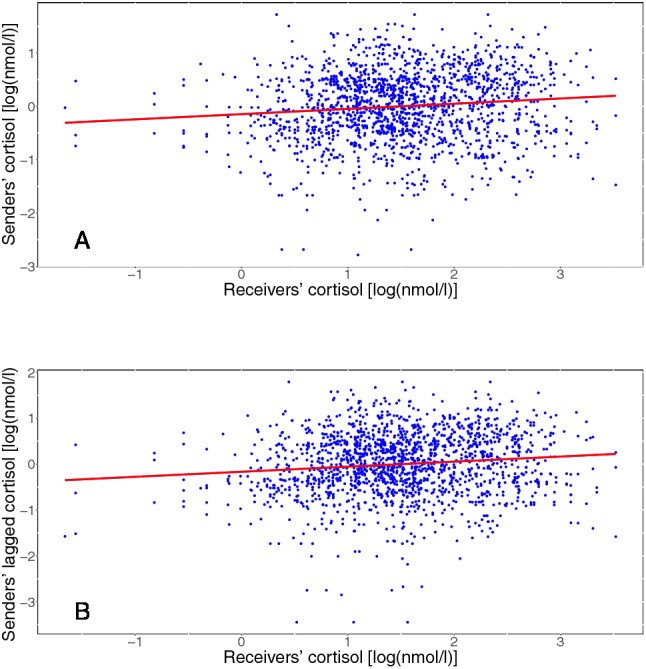
Scatter plot of receivers’ and senders’ concurrent cortisol values (lag size $k = 0$; **A**), and senders’ lagged cortisol values (lag size $k = 1$; **B**) not differentiated by time point or condition. The entire model explained about 94% of the variance in the dependent variable (other independent variables not shown)

**Fig. 4:**
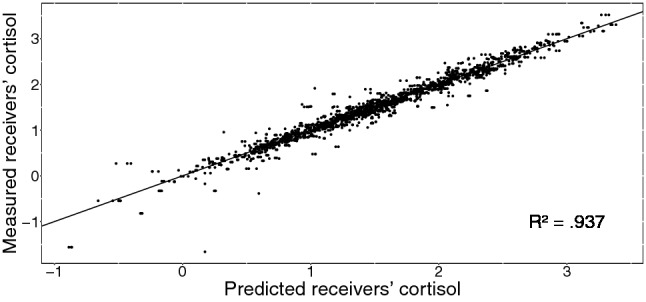
Receivers’ cortisol as predicted by our model versus measured receivers’ cortisol

Time of day during the experiment acted as a significant covariate ($F(1,129) = 39.05, \:p < 0.001$), with higher cortisol values occurring earlier in the day. Gender (in combination with intake of oral contraceptives; levels: male, female with hormonal contraceptives, female without hormonal contraceptives), time since awakening on the day of the experiment, and group size were also tested as potential covariates, but did not improve model fit. The variance explained by the entire model was $R^2 \approx .94$. In comparison to a model without including senders’ cortisol levels, about 4% more variance was explained when including PS in the model. Figure 3 provides a visualization of PS as operationalized in our model. Figure 4 shows the model fit. Table 1 shows $\beta$-coefficients for predictors of the cortisol model. Note that due to a different kind of testing, significance changes for some predictors.

**Table 1:** $\beta$-coefficients with corresponding *t* values for cortisol influence and stability model

Predictor	$\beta$-coefficient	Standard error	DF	*t* value	*p* value
(Intercept)	1.36	0.10	1564	14.00	$<0.001^{*}$
Minute	0.00	0.01	1564	− 0.51	0.608
Minute$^2$	0.00	0.00	1564	0.45	0.653
Minute$^3$	0.00	0.00	1564	− 1.51	0.130
Condition	− 0.11	0.14	129	− 0.77	0.445
Receiver lagged	1.15	0.14	1564	8.19	$<0.001^{*}$
Sender	0.13	0.12	1564	1.09	0.276
Sender lagged	− 0.60	0.15	1564	− 3.96	$<0.001^{*}$
Time of day	− 0.11	0.03	129	− 4.20	$<0.001^{*}$
Minute:condition	0.01	0.01	1564	0.84	0.400
Minute$^2$:condition	0.00	0.00	1564	3.49	$<0.001^{*}$
Minute$^3$:condition	− 0.00	0.00	1564	− 5.01	$<0.001^{*}$
Minute:receiver lagged	− 0.08	0.01	1564	− 8.33	$<0.001^{*}$
Minute$^2$:receiver lagged	0.00	0.00	1564	8.18	$<0.001^{*}$
Minute$^3$:receiver lagged	− 0.00	0.00	1564	− 9.19	$<0.001^{*}$
Condition:receiver lagged	− 1.03	0.17	1564	− 6.03	$<0.001^{*}$
Minute:sender	− 0.02	0.01	1564	− 1.99	0.047$^{*}$
Minute$^2$:sender	0.00	0.00	1564	2.22	0.027$^{*}$
Minute$^3$:sender	0.00	0.00	1564	− 2.35	0.019$^{*}$
Condition:sender	0.12	0.03	1564	4.52	$<0.001^{*}$
Minute:sender lagged	0.05	0.01	1564	4.61	$<0.001^{*}$
Minute$^2$:sender lagged	0.00	0.00	1564	− 4.48	$<0.001^{*}$
Minute$^3$:sender lagged	0.00	0.00	1564	4.24	$<0.001^{*}$
Condition:sender lagged	0.39	0.14	1564	2.76	0.006$^{*}$
Receiver lagged:sender lagged	0.02	0.01	1564	1.91	0.057
Minute:condition:receiver lagged	0.07	0.01	1564	5.91	$<0.001^{*}$
Minute$^2$:condition:receiver lagged	0.00	0.00	1564	− 5.62	$<0.001^{*}$
Minute$^3$:condition:receiver lagged	0.00	0.00	1564	4.88	$<0.001^{*}$
Minute:condition:sender lagged	− 0.03	0.01	1564	− 2.87	0.004$^{*}$
Minute$^2$:condition:sender lagged	0.00	0.00	1564	2.14	0.033$^{*}$
Minute$^3$:condition:sender lagged	0.00	0.00	1564	− 1.55	0.122
Observations	1724				
Log likelihood	− 92.738				
Akaike inf. crit.	267.476				
Bayesian inf. crit.	491.024				

Receivers’ cortisol values are the dependent variable

Predictors are centered

* *p* < 0.05

**Fig. 5:**
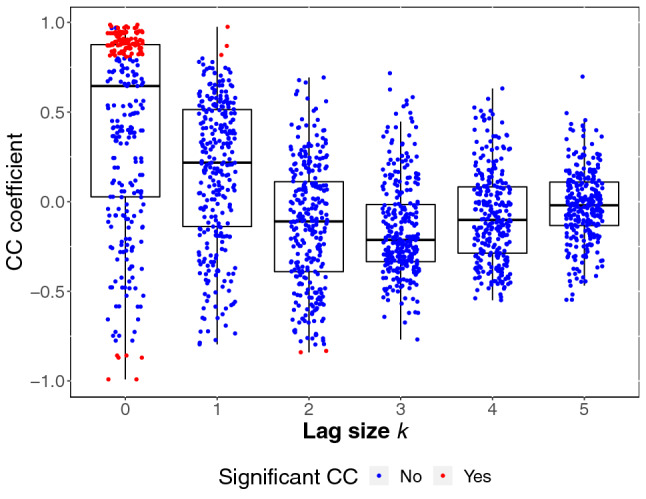
Cross-correlation coefficients (CC coefficients) for pairwise cortisol time series by lag size *k*. The color indicates whether a given CC coefficient exceeded the conventional limit for significance (CCSL). CC coefficients for negative lag sizes are symmetrical to those for positive lag sizes

Influences of lag length on synchrony between group members’ cortisol stress responses were assessed making use of cross-correlation functions. Cross-correlation coefficients are depicted in Fig. 5. While a majority of the CC coefficients indicated a non-significant relationship between participants, there were relatively many pairs of participants who showed a significant CC coefficient at lag size $k = 0$. Some pairs of participants also showed significant negative CC coefficients.

### Alpha-amylase synchrony

**Fig. 6:**
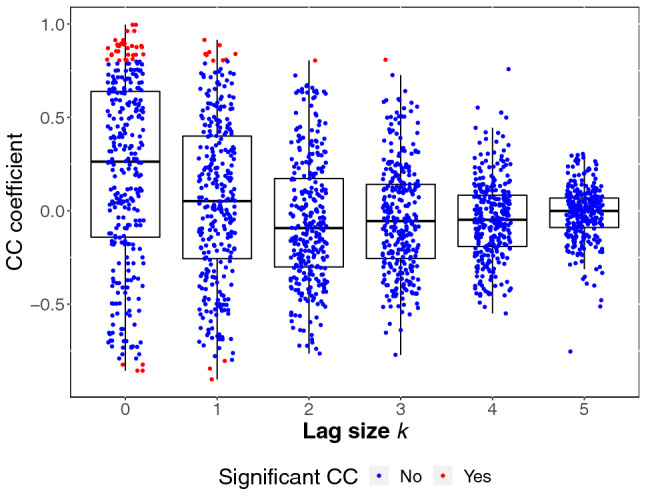
Cross-correlation coefficients (CC coefficients) for pairwise alpha-amylase time series by lag size *k*. The color indicates whether a given CC coefficient exceeded the conventional limit for significance (CCSL). CC coefficients for negative lag sizes are symmetrical to those for positive lag sizes

**Fig. 7:**
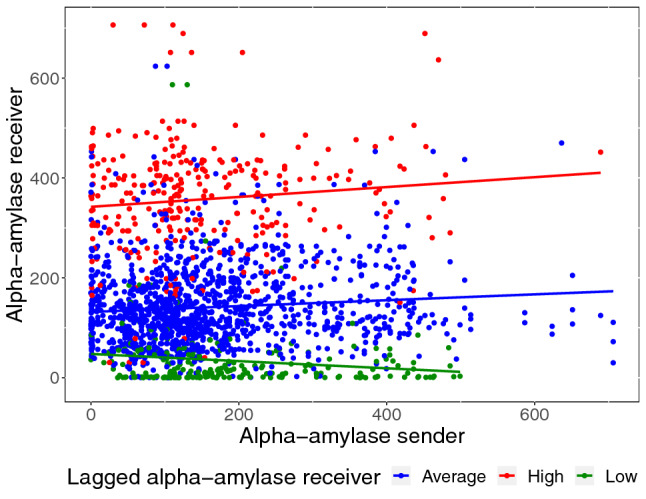
The relationship between senders’ and receivers’ alpha-amylase (physiological influence or synchrony) is moderated by receivers’ own previous stress values

The analysis of alpha-amylase synchrony was conducted analogous to the analysis of cortisol synchrony. Here, a random intercept for each participant, as well as a random linear, quadratic, and cubic slope for each participant improved model fit compared to a basic model. Like for cortisol, we arrived at a final model by adding predictors when they further improved model fit. In this final model, significant quadratic and cubic time effects indicate change in alpha-amylase levels over time (linear effects: $F(1,1664) = 0.45, \:p = .500$; quadratic effects: $F(1,1664) = 48.63, \:p < 0.0001$; cubic effects: $F(1,1664) = 199.13, \:p < 0.001$). While there was no main effect of condition ($F(1,131) = 3.25, \:p = 0.073$), alpha-amylase changed over time depending on condition (linear effects: $F(1,1664) = 1.36, \:p = 0.244$; quadratic effects: $F(1,1664) = 28.29, \:p < 0.001$; cubic effects: $F(1,1664) = 82.50, \:p < 0.001$). Within-person stability was significant ($F(1,1664) = 270.48, \:p < 0.001$), and changed over time (linear effects: $F(1,1664) = 363.63, \:p < 0.001$; quadratic effects: $F(1,1664) = 99.60, \:p < 0.001)$; cubic effects: $F(1,1664) = 34.18, \:p < 0.001$). Stability showed stronger changes over time in the TSST-G condition (linear effects: $F(1,1664) = 32.60, \:p < 0.001$; for quadratic and cubic effects both $p > 0.05$). As for synchrony, there was a main effect of both concurrent ($F(1,1664) = 9.36, \:p = 0.002$) and lagged PS ($F(1,1664) = 13.90, \:p < 0.001$). Interestingly, concurrent PS interacted significantly with receivers’ lagged alpha-amylase ($F(1,1664) = 5.31, \:p = 0.021$), such that PS was higher in receivers who had experienced high physiological stress before (see Fig. 7). Body mass index (BMI) acted as a significant covariate in the final model ($F(1,131) = 35.68, \:p <0.001$), with higher BMI values associated with decreased alpha-amylase values. CC coefficients for alpha-amylase and their significance can be obtained in Fig. 6.

### Exploratory analysis of emotional synchrony

When conducting a stability and influence analysis with subjective stress as the dependent variable, we found different trajectories between experimental conditions, and significant within-person stability. However, participants within the same group did not influence each other significantly, indicating no self-rated emotional synchrony. The final model, when including the covariate gender/hormonal contraception, explained $R^2 \approx .88$ of subjective stress.

## Discussion

In a reanalysis of data from a previous study (Popovic et al. 2020), our aim was to determine whether we could find signs of endocrine synchronization processes dependent on stressor exposure during a paradigm with minimal social interaction. We tested for physiological synchrony between group members exposed to the TSST-G using a stability and influence model approach by Thorson and West (2018). We found that group peers’ cortisol as well as alpha-amylase concentrations predicted an individual’s cortisol or alpha-amylase levels beyond intraindividual stability, indicating PS of both the hypothalamus–pituitary–adrenal (HPA) axis and the sympathetic nervous system. Even though the statistical models tested for the *influence* of the group members, this finding can be interpreted as PS. Physiological influence from one person on another has been found in different contexts before, including TSST settings (Buchanan et al. 2012). Previous studies investigating stress resonance demonstrated that the stress induced in the target of the TSST could create a resonance response in the experimenter (Buchanan et al. 2012), or an observer (Engert et al. 2014). Importantly, in the current study, the results suggest that there is bidirectionality—the model we employed defines each subject in the TSST-G as sender and receiver; thus, individual stress levels affect, and are affected by, other subjects in the vicinity. Bidirectional PS was, therefore, present despite the minimal interaction usually occurring in a TSST-G setting. Specifically, in our experimental setup, we had employed poster boards to visually separate the participants and thus minimize interference. That we were still able to identify signs of PS suggests that this is a robust phenomenon which might occur more frequently than previously thought. Possible mechanisms might be visual cues after the experimental manipulation affecting the endocrine release trajectory, or auditory stimulation (e.g., tone of voice in the TSST-G condition), or olfactory cues (Narciso et al. 2019, e.g., a smell of stress). Which of these factors exactly is responsible for synchronization cannot be assessed by our experimental design. In the future, it would be interesting to measure synchrony when sensory modalities are restricted selectively.

Interestingly, stressor exposure decreased PS of the HPA axis (cortisol PS) compared to a non-stressful control condition, even though participants in the control condition were not exposed to verbal cues from their fellow group members. PS decreased after the completion of the TSST-G (but not after the non-stressful control task). This suggests that PS in cortisol levels is even more present in non-stressful settings despite an effect on both stressful and non-stressful conditions. That would suggest that our ability to respond to, and in part synchronize with, the endocrine activity of those in our vicinity is augmented under non-stressful conditions, i.e., when our HPA axis is operating under baseline conditions. This presents an interesting path for future research as the endocrine synchronization in a non-stressful group setting is, as far as we know, rarely investigated (as is the HPA axis during baseline). It will be interesting to determine if this synchronization depends on additional factors of the group or the individual, and whether it has an effect on the performance of the individual in the group, or the group as a whole. In contrast to cortisol PS, PS of the sympathetic nervous system (alpha-amylase PS) was not influenced by stressor exposure. However, higher previous alpha-amylase in the receiver predicted stronger PS (Fig. 7). Therefore, stress might not dampen but even enhance PS in the sympathetic nervous system. However, alpha-amylase synchrony did not change over time, and might therefore be less influenced by stress in general. Divergences between responses of different stress systems have been reported in the past (Andrews et al. 2013).

This finding is novel and has important repercussions for the use of the TSST-G in stress research. Depending on the experimental question, the researcher should be aware that his or her choice of experimental design will introduce additional variance in the stress responses independent of the individual’s ability to react to a psychosocial stressor. The effect of the group might be negligible if the task is used to induce stress regardless of the magnitude of the individual cortisol stress response—for example, to test a general effect of stress on a subsequent behavioral or cognitive task. If, however, the individual cortisol trajectory in response to stress is the target of the investigation, it might be advisable to switch to the form of single subject TSST, as the TSST-G might create effects that are at least in part due to group composition, and not the individual’s stress perception and processing.

As can be seen in Fig. 5, the CC coefficients for both cortisol and alpha-amylase were negative for some pairs of participants, suggesting that a higher response in the sender led to a lower response in the receiver. Anti-phase synchrony has previously been reported in parent–child dyads (Creavy et al. 2020), and in romantic partners (Reed et al. 2013). For cortisol, significant negative CC coefficients were present in ten pairs, while significant positive CC coefficients were found in 114 pairs, suggesting that it is the exception rather than the norm. All participants who showed anti-phase synchrony were tested in groups of four participants in total, i.e., the biggest group size in our sample. At this point, this is merely an interesting observation however, as statistically, using a model including all pairs of participants, controlling for group size did not improve model fit. Further research will be necessary to determine whether larger groups promote anti-phase synchrony, and if so, which mechanisms are responsible for this connection.

In contrast to physiological synchrony, we could not find emotional synchrony (also referred to as emotional contagion) in our sample. According to the Neurocognitive Model of Emotional Contagion (Prochazkova and Kret 2017), PS precedes, but does not necessarily lead to emotional synchrony. When physiological changes caused by synchronization processes are perceived in the receiver, these changes can be interpreted as emotion, leading to adapting the receiver’s emotional state to that of the sender. However, this interpretation as emotion does not necessarily take place. In our sample, subjective stress as an emotion was independent among group members.

This study has several limitations: First, the original study was not designed with the present research question in mind. Second, while our results demonstrate an interdependence between participants, there is no information about the mechanisms behind this interdependence. Rather than due to PS, interdependence could also originate from situational characteristics that group members had in common at the time of testing; however, all variables known to us (e.g., use of oral contraceptives, time of day) were controlled for in the statistical model and the influence of the group remained significant; at the same time, it is certainly possible that there were additional factors not recorded at the time of testing that influenced the results. Specifically different starting times of experimental sessions can and should be avoided in the future. Moreover, we did not assess known influences on physiological synchrony, like personal characteristics.

These limitations are contrasted by a number of definite strengths of the study: Sample size is rather large and thus allowed for testing of the more complex ‘stability and influence’ model. Both endocrine and subjective stress markers were assessed, and we took special care to test for the influence of potential confounding variables. While there is no unified method to determine PS, stability and influence models as well as related models are accepted methods which have been utilized to assess PS in the previous studies (e.g., Coutinho et al. 2019; Thorson and West 2018).

Taken together, our results show that endocrine synchronization of stress responses before, during, and after the TSST-G might affect individual measurements. We emphasize that these results should be treated as preliminary, as the current study was not designed for that purpose explicitly, and we encourage researchers to test PS under stress more rigorously in future studies. PS is an emerging field of research in the context of collective behavior and its influence on group interactions is not yet fully understood. In future studies employing the TSST-G, depending on the specific experimental design, it should be considered that the use of a group setting will create an additional effect on individuals. In such situations, researchers are encouraged to check for PS in the data, for example by the methods provided here.

In conclusion, PS can be found even in groups without direct personal interaction and might drive physiological activity more than previously expected. However, if participants are exposed to stress, the driving force of PS might lose power. Future research should investigate PS in multiple levels of interaction and under various states of stress to uncover possible mechanisms behind PS as well as influences on and effects of PS.

## Declarations

### Code availability

Code for data analysis will be made available upon request.

## References

[CR1] Andrews J, Ali N, Pruessner JC (2013). Reflections on the interaction of psychogenic stress systems in humans: the stress coherence/compensation model. Psychoneuroendocrinology.

[CR2] Azhari A, Lim M, Bizzego A, Gabrieli G, Bornstein MH, Esposito G (2020). Physical presence of spouse enhances brain-to-brain synchrony in co-parenting couples. Sci Rep.

[CR3] Biesanz JC, Deeb-Sossa N, Papadakis AA, Bollen KA, Curran PJ (2004). The Role of coding time in estimating and interpreting growth curve models. Psychol Methods.

[CR4] Bizzego A, Azhari A, Campostrini N, Truzzi A, Ng LY, Gabrieli G, Bornstein MH, Setoh P, Esposito G (2020). Strangers, friends, and lovers show different physiological synchrony in different emotional states. Behav Sci.

[CR5] Buchanan TW, Bagley SL, Stansfield RB, Preston SD (2012). The empathic, physiological resonance of stress. Soc Neurosci.

[CR6] Cohen S, Kamarck T, Mermelstein R (1983). A global measure of perceived stress. J Health Soc Behav.

[CR7] Coutinho J, Oliveira-Silva P, Fernandes E, Gonçalves OF, Correia D, Perrone Mc-Govern K, Tschacher W (2019). Psychophysiological Synchrony during verbal interaction in romantic relationships. Fam Process.

[CR8] Creavy KL, Gatzke-Kopp LM, Zhang X, Fishbein D, Kiser LJ (2020). When you go low, I go high: negative coordination of physiological synchrony among parents and children. Dev Psychobiol.

[CR9] Danyluck C, Page-Gould E (2019). Social and physiological context can affect the meaning of physiological synchrony. Sci Rep.

[CR10] Dean RT, Dunsmuir WT (2016). Dangers and uses of cross-correlation in analyzing time series in perception, performance, movement, and neuroscience: the importance of constructing transfer function autoregressive models. Behav Res Methods.

[CR11] Dimitroff SJ, Kardan O, Necka EA, Decety J, Berman MG, Norman GJ (2017). Physiological dynamics of stress contagion. Sci Rep.

[CR12] Dressendörfer RA, Kirschbaum C, Rohde W, Stahl F, Strasburger CJ (1992). Synthesis of a cortisol-biotin conjugate and evaluation as a tracer in an immunoassay for salivary cortisol measurement. J Steroid Biochem Mol Biol.

[CR13] Ellamil M, Berson J, Margulies DS (2016). Influences on and measures of unintentional group synchrony. Front Psychol.

[CR14] Enders CK, Tofighi D (2007). Centering predictor variables in cross-sectional multilevel models: a new look at an old issue. Psychol Methods.

[CR15] Engert V, Plessow F, Miller R, Kirschbaum C, Singer T (2014). Cortisol increase in empathic stress is modulated by emotional closeness and observation modality. Psychoneuroendocrinology.

[CR16] Finch WH, Bolin JE (2017). Multilevel modeling using Mplus. CRC Press, Boca Raton..

[CR17] Gordon I, Gilboa A, Cohen S, Milstein N, Haimovich N, Pinhasi S, Siegman S (2020). Physiological and Behavioral synchrony predict group cohesion and performance. Sci Rep.

[CR18] Gvirts HZ, Perlmutter R (2020). What guides us to neurally and behaviorally align with anyone specific? A neurobiological model based on fNIRS hyperscanning studies. Neuroscientist.

[CR19] Järvelä S, Kätsyri J, Ravaja N, Chanel G, Henttonen P (2016). Intragroup Emotions: physiological linkage and social presence. Front Psychol.

[CR20] Kirschbaum C, Pirke KM, Hellhammer DH (1993). The Trier social stress test-a tool for investigating psychobiological stress responses in a laboratory setting. Neuropsychobiology.

[CR21] Koban L, Ramamoorthy A, Konvalinka I (2019). Why do we fall into sync with others? Interpersonal synchronization and the brains optimization principle. Soc Neurosci.

[CR22] Konvalinka I, Xygalatas D, Bulbulia J, Schjødt U, Jegindø EM, Wallot S, Van Orden G, Roepstorff A (2011). Synchronized arousal between performers and related spectators in a fire-walking ritual. Proc Natl Acad Sci USA.

[CR23] Lüdecke D, Makowski D, Waggoner P, Patil I (2020) Performance: assessment of regression models performance. https://CRAN.R-project.org/package=performance, R package version 0.4.7

[CR24] McNaughton KA, Redcay E (2020). Interpersonal Synchrony in autism. Curr Psychiatry Rep.

[CR25] Miller R, Stalder T, Jarczok M, Almeida DM, Badrick E, Bartels M, Boomsma DI, Coe CL, Dekker MC, Donzella B, Fischer JE, Gunnar MR, Kumari M, Lederbogen F, Power C, Ryff CD, Subramanian SV, Tiemeier H, Watamura SE, Kirschbaum C (2016). The CIRCORT database: reference ranges and seasonal changes in diurnal salivary cortisol derived from a meta-dataset comprised of 15 field studies. Psychoneuroendocrinology.

[CR26] Narciso D, Bessa M, Melo M, Vasconcelos-Raposo J (2019) Virtual reality for training-the impact of smell on presence, cybersickness, fatigue, stress and knowledge transfer. ICGI 2019—Proceedings of the international conference on graphics and interaction, 115–121, 10.1109/ICGI47575.2019.8955071

[CR27] Palumbo RV, Marraccini ME, Weyandt LL, Wilder-Smith O, McGee HA, Liu S, Goodwin MS (2017). Interpersonal Autonomic physiology: a systematic review of the literature. Personal Soc Psychol Rev.

[CR28] Pinheiro J, Bates D, DebRoy S, Sarkar D, R Core Team (2019) nlme: linear and nonlinear mixed effects models. https://CRAN.R-project.org/package=nlme, R package version 3.1-143

[CR29] Popovic NF, Bentele UU, Pruessner JC, Moussaïd M, Gaissmaier W (2020). Acute stress reduces the social amplification of risk perception. Sci Rep.

[CR41] Pruessner JC, Kirschbaum C, Meinlschmid G, Hellhammer DH (2003) Two formulas for computation of the area under the curve represent measures of total hormone concentration versus time-dependent change. Psychoneuroendocrinology 28(7):916–931. 10.1016/S0306-4530(02)00108-7 10.1016/s0306-4530(02)00108-7 12892658

[CR30] Prochazkova E, Kret ME (2017). Connecting minds and sharing emotions through mimicry: a neurocognitive model of emotional contagion. Neurosci Biobehav Rev.

[CR31] R Core Team (2019) R: a language and environment for statistical computing. R Foundation for Statistical Computing, Vienna, Austria, https://www.R-project.org/

[CR32] Reed RG, Randall AK, Post JH, Butler EA (2013). Partner influence and in-phase versus anti-phase physiological linkage in romantic couples. Int J Psychophysiol.

[CR33] Rosenberg M (1965). Society and the adolescent self-image.

[CR34] RStudio Team (2020) RStudio: integrated development environment for R. RStudio, PBC., Boston, MA, http://www.rstudio.com/

[CR35] Shamay-Tsoory SG, Saporta N, Marton-Alper IZ, Gvirts HZ (2019). Herding brains: a core neural mechanism for social alignment. Trends Cogn Sci.

[CR36] Thorson KR, West TV (2018). Physiological linkage to an interaction partner is negatively associated with stability in sympathetic nervous system responding. Biol Psychol.

[CR37] Thorson KR, West TV, Mendes WB (2018). Measuring physiological influence in dyads: a guide to designing, implementing, and analyzing dyadic physiological studies. Psychol Methods.

[CR38] von Dawans B, Kirschbaum C, Heinrichs M (2011). The Trier Social Stress Test for Groups (TSST-G): a new research tool for controlled simultaneous social stress exposure in a group format. Psychoneuroendocrinology.

[CR39] Vors O, Marqueste T, Mascret N (2018). The trier social stress test and the trier social stress test for groups: qualitative investigations. PLoS One.

[CR40] Wróbel M, Królewiak K (2017). Do we feel the same way if we think the same way? shared attitudes and the social induction of affect. Basic Appl Soc Psychol.

